# Dynamic task offloading edge-aware optimization framework for enhanced UAV operations on edge computing platform

**DOI:** 10.1038/s41598-024-67285-2

**Published:** 2024-07-16

**Authors:** B. Suganya, R. Gopi, A. Ranjith Kumar, Gavendra Singh

**Affiliations:** 1 Faculty of Artificial Intelligence & Data Science, Sri Ramakrishna Engineering College, Coimbatore, Tamil Nadu 621112 India; 2 Faculty of Computer Science & Engineering, Dhanalakshmi Srinivasan Engineering College, Perambalur, Tamil Nadu 621212 India; 3 Faculty of Computer Science and Engineering, Lovely Professional University, Phagwara, 144001 Punjab India; 4 Faculty of Software Engineering, College of Computing and Informatics, Haramaya University, Dire Dawa, P.O. Box 138, Ethiopia

**Keywords:** Optimization, Artificial intelligence, Edge computing, Performance, Offloading, Computer science, Computational science, Information technology

## Abstract

Resource optimization, timely data capture, and efficient unmanned aerial vehicle (UAV) operations are of utmost importance for mission success. Latency, bandwidth constraints, and scalability problems are the problems that conventional centralized processing architectures encounter. In addition, optimizing for robust communication between ground stations and UAVs while protecting data privacy and security is a daunting task in and of itself. Employing edge computing infrastructure, artificial intelligence-driven decision-making, and dynamic task offloading mechanisms, this research proposes the dynamic task offloading edge-aware optimization framework (DTOE-AOF) for UAV operations optimization. Edge computing and artificial intelligence (AI) algorithms integrate to decrease latency, increase mission efficiency, and conserve onboard resources. This system dynamically assigns computing duties to edge nodes and UAVs according to proximity, available resources, and the urgency of the tasks. Reduced latency, increased mission efficiency, and onboard resource conservation result from dynamic task offloading edge-aware implementation framework (DTOE-AIF)'s integration of AI algorithms with edge computing. DTOE-AOF is useful in many fields, such as precision agriculture, emergency management, infrastructure inspection, and monitoring. UAVs powered by AI and outfitted with DTOE-AOF can swiftly survey the damage, find survivors, and launch rescue missions. By comparing DTOE-AOF to conventional centralized methods, thorough simulation research confirms that it improves mission efficiency, response time, and resource utilization.

## Introduction

The application of AI to improve the operations of UAVs at the network's edge presents several opportunities and potential dangers^1^. As one of the most significant challenges, the complex connection between the need for data processing and the capacity to make real-time judgments is particularly challenging. UAVs generate enormous amounts of data, urgently needing to be assessed and addressed to ensure optimal operation^2^. It is possible that normal cloud-based solutions could result in latency issues due to the amount of time it takes to transmit data to remote servers for processing. This latency may hamper critical decision-making processes, particularly in dynamic circumstances requiring prompt reactions^3^. It is important to note that UAVs have limited computer capability, which restricts the complexity of AI algorithms that may be utilized for real-time processing. Optimization becomes even more challenging when balancing the computational tasks performed onboard and those being offloaded to edge computing nodes^4^. There is an additional difficulty added by the fact that data privacy and security during transmission and processing at the edge are concerned. Despite these obstacles, edge computing that is powered by AI has the potential to enhance the operations of UAVs^5^ by enabling faster decision-making, improved resource use, and higher autonomy. To develop solutions that are effectively scalable and tailored to the uses of UAVs, it is necessary to adopt new methods of thinking about these issues. Such issues use advancements in AI, edge computing, and communication technology^6^. Cooperation between researchers, industry stakeholders, and regulatory authorities is required to overcome these obstacles and fully exploit the potential of edge computing driven by AI for optimizing UAV operating procedures^7^.

Current approaches to enhancing UAV operations through AI-driven edge computing encompass various ways to improve performance and reduce issues. The practice of task offloading, which involves moving computationally demanding activities to edge computing nodes that are located close to the operational region of the UAV, is a typical solution that is used to reduce latency and conserve onboard resources^8^. Using machine learning algorithms for real-time data processing is one alternative strategy that may empower UAVs to make autonomous judgments in response to sensor inputs and ambient variables better^9^. Using edge caching techniques makes it possible to store data that is accessed frequently locally, hence further reducing latency and the requirement for data transmission. Nevertheless, these approaches face various challenges, notwithstanding their effectiveness^10^. Especially in dynamic and diverse situations, it is difficult to guarantee that UAVs and edge computing nodes can interact without interruptions; this is especially true^11^. In addition, optimizing resource management and task allocation to satisfy performance requirements while balancing the amount of computing effort and energy consumed is not easy^12^. Additional obstacles occur due to the requirement to transfer and manage sensitive data at the network's edge while simultaneously addressing concerns regarding privacy and security. Another significant consideration is how AI algorithms interact with different UAV platforms and edge computing architectures regarding scalability and interoperability^13^. It is necessary for researchers from various domains, such as AI, Edge Computing (EC), communication, and cybersecurity, to collaborate to address these challenges. Academic institutions, industries, and government agencies must collaborate to set guidelines for optimizing UAV operations through edge computing driven by AI^14^. Traditional centralized processing architectures in UAV operations encounter latency, bandwidth limits, scalability issues, and limited onboard computational resources. The research addresses these issues^15^. Goals include mission success, resource optimization, and timely data acquisition with AI-powered edge computing.

For real-time data processing and analysis, edge AI means deploying AI models and algorithms directly on local edge devices like sensors or Internet of Things (IoT) devices rather than relying on cloud infrastructure. Edge AI, frequently referred to as "AI on the edge," uses AI in conjunction with edge computing to run machine learning tasks directly on connected edge devices. Data can be maintained near the device using edge computing and processed on the network edge using AI algorithms, independent of whether the device has an internet connection or otherwise. It enables for millisecond-level data processing, resulting in immediate response.

The Dynamic Job Offloading Edge-Aware Optimization Framework optimizes job allocation in real time by combining cutting-edge AI algorithms and edge computing approaches. In its most basic form, RL agents make a dynamic choice on whether or not to offload work to edge devices or the cloud by utilizing regularly updated predictions from DL models. Federalized learning is used to train these models to safeguard the data's confidentiality and increase the process's effectiveness. Graph Neural Networks (GNNs) simplify scheduling and resource allocation by mimicking the whole network topology. This design is improved further by GNNs. Using edge orchestration and load-balancing strategies, workloads may be distributed consistently. Reducing latency and bandwidth usage with real-time analytics and edge caching assistance is possible. This integrated approach, which enables the framework to adapt dynamically to constantly changing network conditions, ensures that tasks are executed efficiently and resources are utilized effectively across the edge network.

The DTOE-AOF has several primary objectives, one of which is to address several significant gaps in the state of the art of edge computing. In dynamic environments, conventional approaches, such as heuristic methods and static task offloading, usually perform suboptimally due to their lack of flexibility and adaptability. Both of these methods are examples of traditional methodology. Even though there has been considerable investigation into machine learning and reinforcement learning techniques, these approaches are confronted with several challenges, including centralized data processing, privacy issues, and high computation needs. Additionally, it has a restricted capacity for scaling up. The DTOE-AOF utilizes several cutting-edge AI techniques. These techniques include federated learning-trained DL models, which enhance efficiency and privacy, and reinforcement learning, which allows for real-time adaptability. Via the utilization of GNNs to explain the network architecture, as well as via edge management and load balancing techniques, the system guarantees the optimal and scalable allocation of the resources required. Edge caching and real-time analytics, which both reduce latency and bandwidth consumption, make it possible to offload real-time tasks in a way that is both efficient and adaptive. It is a robust system that can adapt to changing network situations, scale effectively, integrate diverse data sources, protect user privacy, and optimize real-time offloading decisions. It is all made possible by the all-encompassing design of DTOE-AOF.

### Problem definition

Latency, bandwidth constraints, and scalability problems are problems that conventional centralized processing architectures encounter. An opportunity to overcome these obstacles and realize UAVs' full potential has arisen with the introduction of AI-powered edge computing. Concerns with limited onboard computational resources, unpredictable climatic conditions, and severe latency requirements are some obstacles to deploying UAVs in real-world applications.

Specifically, it addresses challenges with self-driving vehicles, smart cities, and industrial IoT that current approaches fail to address in edge computing environments completely. Low-latency states and real-time data processing are essential for these kinds of circumstances. Separating data sources and processing centres causes delays in traditional cloud-based methods. Due to rapid fluctuations in network situations and resource availability, static offloading solutions cannot adapt to these environments. However beneficial, heuristic approaches generally require enormous processing capacity and fail to perform effectively across many devices.

### Objectives

- Optimization of UAV operations is proposed via the DTOE-AOF. This framework dynamically distributes edge nodes and UAVs computational tasks based on proximity, resources, and task urgency.
- Integrating AI algorithms with edge computing reduces latency, boosts mission efficiency, and conserves onboard resources.
- The research will show DTOE-AOF's versatility and usefulness in precision agriculture, emergency management, infrastructure inspection, and monitoring.
- If UAVs have DTOE-AOF, precision agriculture can use data-driven decision-making, disaster management can use quick surveys, and infrastructure inspections can be more thorough.
- The research attempts to confirm DTOE-AOF's improved mission efficiency, response time, and resource utilization through simulation and comparison with standard centralized approaches.

The remainder of the research paper is organized as follows: “Literature review” provides a literature analysis on optimizing UAV operations through AI to improve performance at the border. “Proposed method” focuses on the mathematical aspects of the DTOE-AOF. A detailed description of the experiment's findings, analysis, and comparisons to prior methodologies are included in “Results and discussion”. The results are summed up in “Conclusion”.

## Literature review

Several industries are seeing profound shifts due to the confluence of state-of-the-art technologies like the IoT, DL, optimization methods, edge computing, and beyond 5G (B5G)/6G wireless networks. One area where this is taking off is UAVs. This literature review explores new developments and methods in these areas, focusing on creative strategies and the practical effects of these developments.

### (a) Review on UAV

Using AI built into UAVs, Koubaa et al.^26^ present a cloud-edge hybrid system (C-EHS)that can do remote sensing. The onboard AI system, AERO, integrates object identification and tracking to achieve precision and transmit data in real-time.

Lins et al.^27^ put forward the ideas of System Intelligence (SI) and Edge Intelligence (EI) to use 5G networks for UAV-based SAR (UAV-SAR)operations. Critical to mission efficiency, it provides a virtualized testbed to show how DNN partitioning affects communication costs and latency. Ijaz H. et al.^28^ suggest a UAV-assisted edge computation framework, UAV-ECF, for real-time catastrophe scenario categorization, compressing CNN models for onboard GPUs. The results reveal a reduction of 84% in model size and an increase of 99% in throughput without sacrificing accuracy.

Addressing the issue of deploying bulky models on resource-constrained devices, Surianarayanan et al.^29^ investigate AI model optimization approaches. An AI model optimization framework is needed to get insights into edge intelligence applications in real-time. Palossi et al. present PULP-Frontnet^30^, a deep neural network (DNN) for UAVs that uses vision to estimate human poses in real-time. Compared to optimal sensor setups, the results show that autonomous navigation uses very little energy while providing great accuracy.

### (b) Optimization using AI

The paper introduces methodologies and tools for optimizing vision-based CNNs on ULP processors for nano-UAVs. Deploying PULP-Dronet improves memory efficiency and speed, enhancing obstacle avoidance, free flight, and lane following while conserving power. To improve the IoT, Xu et al.^31^ investigated how AI and backscatter communication (BC) might work together. It provides an overview of current developments, such as AI algorithms for security, channel estimate, and signal detection. It also delves into potential future uses in B5G/6G technologies, outlining opportunities and threats. Using IoT devices, robotic drones operated by AI are investigated by Jat et al.^32^. concerning the COVID-19 response. The topics covered are improving efficiency in pandemic conditions, using edge computing for fast data analysis, and reviewing literature for future research paths.

For heterogeneous computing systems involving UAVs, Kim et al.^33^ present a computational offloading scheme that considers energy consumption and fairness. The solution improved energy consumption fairness by up to 120% compared to the prior methodology, all recognitions to a genetic algorithm.

The article by Miya et al.^34^ delves into how the IoT is progressing towards intelligent services and how upgraded wireless networks, such as 6G, are necessary. A combination of AI with quantum communications is suggested to fulfil the needs of upcoming applications by Miya et al.^35^.

### (c) Edge computation

Recent advances in Reinforcement Learning (RL) for Multi-UAV Wireless Networks (MUWNs) are reviewed by Bai et al.^36^, who draw attention to open difficulties in the field and describe RL applications such as data access, resource allocation and trajectory planning. Optimal offloading and task prediction in Fog Computing (FC) networks are proposed by Jana et al.^37^ using an analytical technique. Concerning load balancing and reaction time, the LSTM-GWO approach surpasses the current ACO and PSO approaches.

Smart city and society management is the focus of Heidari et al.'s^38^ investigation into using AI, ML, and DL approaches in conjunction with the IoT, IoD, and IoV. The article discusses recent advancements, advantages, and disadvantages, emphasizing smart traffic and energy management. The findings emphasize the importance of ML approaches like CNN and LSTM and the prevalence of their use in smart city applications, where accuracy is paramount. Based on the results of these investigations, Python is the language used in computer programming.

Pandey et al.^39^ present a new way to evaluate Mobile Edge Computing (MEC) in 5G networks using simulated data. It allows for rapid evaluations of MEC performance and optimization of resources by properly capturing spatio-temporal trends through advanced modelling approaches.

For wireless connectivity beyond 5G, the DEDICAT 6G project, which the European Union supports, aims to provide a smart and environmentally friendly platform (Stavroulaki et al. 2018^40^). Utilizing robots, linked vehicles, and drones enhances task execution speed, energy efficiency, and latency while investigating dynamic coverage extensions. Innovative interfaces, such as smart glasses, facilitate human–machine contact, and the project deals with privacy, security, and trust assurance. Network load balancing, resource allocation, coverage extension, security, and human–machine applications are the goals of the demonstrations and testing in four illustrative use cases.

Modern AI systems that rely on big data analytics and DL have high communication and determine cost, which results in high energy consumption, network congestion, and privacy leaks during training and inference. Edge AI was a game-changing innovation for 6G networks that improved their efficacy, privacy, security, and efficiency by introducing model training and inference capabilities to the network's edge^42^. This enables seamless integration of sensing, communication, analysis, and intelligence. Using decentralized machine learning models and integrated designs for wireless communication techniques, the author aims to create trustworthy and scalable edge AI systems in this work. This paper discusses new wireless network design concepts, optimization methodologies for service-driven resource allocation, and an end-to-end system architecture that supports edge AI.

IoT and autonomous system technologies are an appropriate match for edge information processing methods and approaches, but many problems still require addressing. This paper aims to analyze these emerging multimedia and edge information processing paradigms from various technological perspectives. These viewpoints include AI-powered multimedia analytics on the edge, intelligent edge multimedia streaming^43^, AI-powered multimedia caching on the edge, AI-powered multimedia services for the edge, and AI-powered hardware and devices for intelligent multimedia on the edge. The study addresses various AI and ML enablers for multimedia and edge data processing.

The summary of the findings is given in Table 1.

**Table 1:** Comparison of the existing methods.

S. no.	Literature	Method	Advantages	Limitations
1	^26^	Cloud-edge hybrid system (C-EHS)	Precision in remote sensing, real-time data transmission, integration of object identification and tracking	Reliance on cloud connectivity, potential latency issues
2	^27^	System intelligence (SI) and edge intelligence (EI) for UAV-based SAR operations	Utilization of 5G networks, virtualized testbed for DNN partitioning analysis, and efficiency in mission-critical tasks	Communication costs, latency issues, dependency on network stability
3	^28^	UAV-assisted edge computation framework (UAV-ECF)	Real-time catastrophe scenario categorization, significant reduction in model size, increased throughput without accuracy loss	Initial setup and configuration complexity, potential hardware compatibility issues
4	^29^	AI model optimization approaches	Insights into edge intelligence applications, optimization of AI models for resource-constrained devices	Complexity in implementation, potential trade-offs between optimization and model accuracy
5	^30^	PULP-Frontnet	Real-time human pose estimation, energy-efficient autonomous navigation, and high accuracy compared to optimal sensor setups	Dependency on vision quality, potential computational overhead
6	^31^	Optimization of vision-based CNNs on ULP processors for nano-UAVs	Memory efficiency improvement, enhanced obstacle avoidance, free flight, and lane following power conservation	Hardware compatibility, potential performance trade-offs
7	^32^	Integration of AI and backscatter communication for IoT enhancement	Advancement in AI algorithms for security, channel estimation, and signal detection, potential improvement in IoT functionalities	Complexity in integration, potential security vulnerabilities
8	^33^	Computational offloading scheme for heterogeneous computing systems involving UAVs	Improved energy consumption fairness, consideration of energy efficiency and fairness, utilization of genetic algorithm for optimization	Complexity in algorithm design, potential scalability issues
9	^34^	Progression of IoT towards intelligent services and the necessity of upgraded wireless networks (6G)	Advancement in wireless communication technologies, potential for intelligent IoT services	Dependency on infrastructure upgrades, potential compatibility issues
10	^35^	Integration of AI with quantum communications	Potential enhancement in communication security and efficiency	Complexity in implementation, current limitations in quantum communication technologies
11	^36^	Reinforcement learning for multi-UAV wireless networks (MUWNs)	Advancement in data access, resource allocation, and trajectory planning, potential for optimized UAV network operations	Complexity in algorithm design, potential scalability issues
12	^37^	Optimal offloading and task prediction in fog computing networks using LSTM-GWO approach	Improved load balancing and reaction time, surpassing current approaches like ACO and PSO	Complexity in algorithm design, potential computational overhead
13	^38^	Utilizing AI, ML, and DL approaches in smart city and society management	Advancement in smart traffic and energy management, emphasis on accuracy through ML approaches like CNN and LSTM	Data privacy concerns, potential bias in AI algorithms
14	^39^	Evaluation of MEC in 5G networks using simulated data	Rapid evaluation of MEC performance, optimization of resources through spatiotemporal trend analysis	Reliance on simulated data accuracy, potential discrepancy with real-world scenarios
15	^40^	DEDICAT 6G project for smart wireless connectivity beyond 5G	Enhanced task execution speed, energy efficiency, and latency, investigation of dynamic coverage extensions, human–machine interaction enhancement through innovative interfaces	Dependency on project funding and support, potential regulatory hurdles

These studies aim to improve disaster management, healthcare monitoring, smart city infrastructure, and wireless communication networks by developing technology-driven solutions to tackle complicated problems.

Based on the analysis, there are numerous problems with present models in attaining a high mission efficiency, response time, and resource utilization. Hence, this study proposes the Dynamic Task Offloading Edge-Aware Optimization Framework (DTOE-AOF) for UAV operations optimization.

## Proposed method

### UAV with DTOE-AOF implementation

Improving efficiency, decreasing latency, and making the most of available resources are becoming increasingly important in the ever-changing world of UAV operations. Latency, bandwidth limitations, and scalability problems are problems with traditional central processing methods. This study presents the DTOE-AOF to address these issues. With AI-powered decisions and edge computing buildings, DTOE-AOF may dynamically assign computing jobs to UAVs and edge nodes according to proximity, available resources, and the urgency of the work. With its groundbreaking method, DTOE-AOF is poised to revolutionize various programs, from agricultural precision to emergency management, by reducing latency, increasing mission efficiency, and conserving resources.

**Figure 1:**
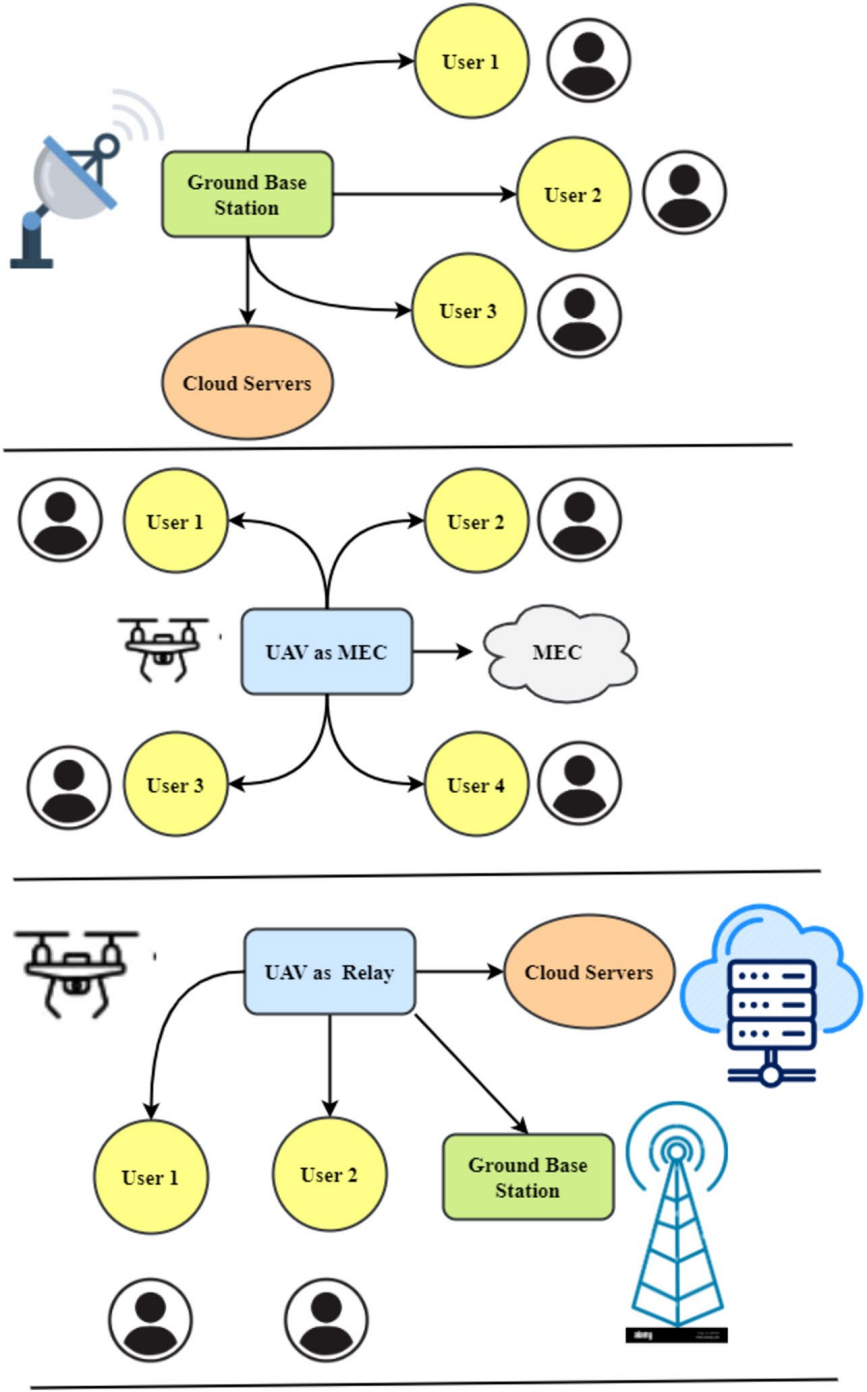
Architecture facilitated by UAVs.

MEC is commonly recognized as a basic technology for various next-generation IoT applications. Due to their versatility and ease of deployment, UAVs can provide edge computing services. UAV-enabled MEC designs may be categorized and applied. A UAV may be a relay, IoT node, or mobile EC server. First, UAVs may act as mobile nodes by sending their computing power to a MEC server. As the MEC, the UAV may monitor a cluster. The third usage of UAVs is linking mobile end nodes to MEC servers. Figure 1 shows that the UAV may submit memory- and processing-intensive operations to an MEC server as a dedicated user. Complex computational jobs may be too difficult for UAVs' processing, memory, and battery life. Moving data processing to the ground-based MEC server may extend its battery life. Figure 1 depicts an alternate scenario in which the UAV flies with the MEC server to assist ground users with computing once they offload their jobs to it. In the third alternative, Fig. 1, the UAV acts as a central relay to let mobile users transmit their computing operations to an MEC server.

UAV-enabled MECs' design provides IoT devices with reliable, low-latency services. Still, turmoil abounds. Airborne data security, storage, administration, and UAV networking are complex. UAV mobility complicates communication, requiring greater ground-based user-UAV cooperation. The low battery power of UAVs is another challenge. More power is required for onboard computations before hovering, accelerating/decelerating, climbing/descending. To start planning for better energy and resource management immediately. Another challenge when developing UAV-enabled MEC systems for computing work is integrated route engineering. Predicting and tracking mobile user behaviour is essential for the optimum offloading of computing tasks and timely communication of computing results to consumers. Many UAV MEC services require greater trajectory design consideration. UAV blockchain integration is difficult^16^. UAVs pose privacy issues, air traffic law violations, quantum attacks, machine learning, and algorithmic attacks alone.

1 $$L= \frac{\beta .\left(\alpha . TT+ \delta . PT+ \vartheta . PT\right)}{\sigma + \rho +N \pi }$$

Equation (1) makes the examination of latency $L$ during UAV operations more complicated. A subtle change in the importance of specific components within the latency equation is made possible by the weighting $\text{} \beta$, $\alpha . TT$, $\delta . PT$, and $\vartheta . PT$. The parameter $\sigma$ captures the fluctuating character of the network's capacity by modulating the influence of Wavelength on the overall delay, adding further complexity. Furthermore, the complicated network $N$ environment is reflected in the intricate impact of the regulators $\rho$ and $\pi$ on Standing in line Time and Connection Overhead, respectively. This all-encompassing equation adequately captures the intricate interplay of factors, which provides a thorough comprehension of delays in UAV operations.

2 $$T= \frac{\theta . \left(\mu . DS+\delta . OU+\tau .ET\right)}{XE. \sigma }+BG. \omega +TS. \varphi$$

Equation (2) provides a complex formula that explores the main factors that contribute to the scalability of a system, capturing the complexities of scalability where $T$ in UAV operations. Various weights $\theta$, $\mu$, $\delta$, and $\delta$ allow for subtle modifications to the relevance of Computational Resources $DS$, Network Throughput $OU$, and Data Storage $ET$. The parameter $\omega$ enhances the overall scalability by adjusting the impact of Workload Distribution $BG$. In addition, the Adaptability Factor $XE$ and System Redundancy $TS$ dynamics are elaborately captured by the modifiers $\omega$ and $\varphi$. This simple formula gives a full picture of the complex interrelationships that determine the scalability of UAV operations, shedding light on the dynamics of redundancy and flexibility in the system^17^.

3 $$P= \frac{\beta . N{F}^{\gamma }+\alpha .S{U}^{\theta }}{\delta .S{T}^{\rho }}+{\sum }_{j=1}^{o}{\tau }_{j}. \left({\mu }_{j}.{BJ}_{j}^{{L}_{j}}.{CE}_{j}^{{\sigma }_{j}}.{TO}_{j}^{{\tau }_{j}}\right)$$

Equation (3) incorporates a complicated model for evaluating the performance $P$ of UAV operations. The importance of mission efficiency where $N{F}^{\gamma }$, response time $S{U}^{\theta }$, and resource utilisation $S{T}^{\rho }$ are represented by the weighted coefficients $\beta$, $\alpha ,$ and $\delta$, respectively. Equational intricacy is enhanced by the inclusion of non-linear exponents such as ${TO}_{j}^{{\tau }_{j}}$, $\mu$, and ${\sigma }_{j}$, which magnifies the influence of these basic elements on overall performance by capturing complex dependencies. Furthermore, by taking into account further elements like AI integration, edge computing $CE$ capabilities, and dynamic job offloading methods, the terms involved add a greater degree of complexity.

### UAV with MEC implementation

MEC solves the mobile IoT device resource and time issue. Backhaul congestion and network latency may be reduced with additional CC products. UAVs with data storage, processing, and communication may deploy MEC servers at network edges^18^. In this design, low-powered IoT devices may outsource computation to UAVs with MEC servers via line-of-sight communication. The system must forecast tasks, deploy UAVs, organize users, analyze signals, and allocate cooperative resources, among other challenges. The EC architecture suggests that transportable and adaptable UAVs will provide decentralized solutions. The flying edge architecture's increased CC capabilities are ideal for real-time, latency-sensitive IoT applications. Moving computation from data centres to IoT devices improves real-time administration and decision-making with lower latency. An IoT system's many endpoints flood peripheral devices with data.

Data organization and processing are needed for automated maintenance, self-monitoring, and prediction. Due to the memory and processing power gap between EC endpoints and centralized cloud servers, certain AI systems cannot analyze edge data. Any resilience-focused AI approach must prepare for memory and processing capacity restrictions. Decentralized resource distribution allows EC to fulfil client requests quicker than a CC, even with minimum processing^19^. Work scheduling, resource allocation, and offloading issues may dramatically impact performance. Over several decades, AI has been more popular for networking difficulties. ML is used in various areas, including networking, for its decision-making and interaction abilities. It may enhance network performance in numerous areas, including resource allocation, traffic classification and prediction, congestion control, and routing.

**Algorithm 1:**
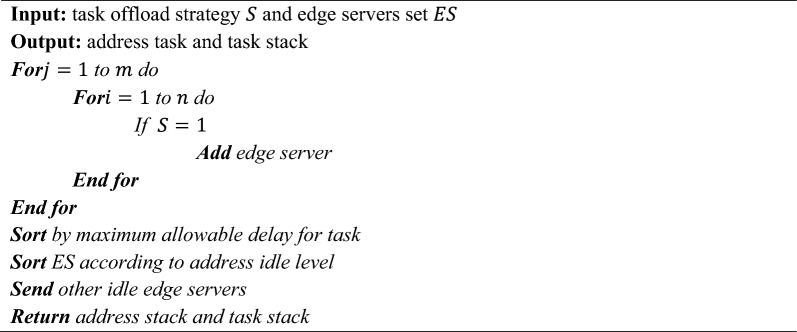
Resource allocation based on the proposed DTOE-AOF.

Algorithm 1 shows the Resource Allocation based on the proposed DTOE-AOF. Developing a resource allocation strategy aims to increase the likelihood of the task's success. Finding the best way to reduce latency in cloud and edge-cloud collaboration systems requires a combination of communication technologies and computer resource allocation. This research developed a distributed computing-based offloading strategy that can adapt to changing user loads and achieve outstanding computing offloading capabilities. In an environment with many channels of wireless interference, our research found the best solution to the issue of offloading resources from the edge cloud to multiple users. Allotting resources among edge servers is modelled in this research. Time is of the essence for crowd activities as well since edge servers have limited processing power and load capacity and must finish computing jobs within a certain time frame. As a result, this article classifies the tasks by priority and saves them in the task stack, equivalent to the address stack in the edge server, based on the maximum permissible delay of the tasks^20^.

**Figure 2:**
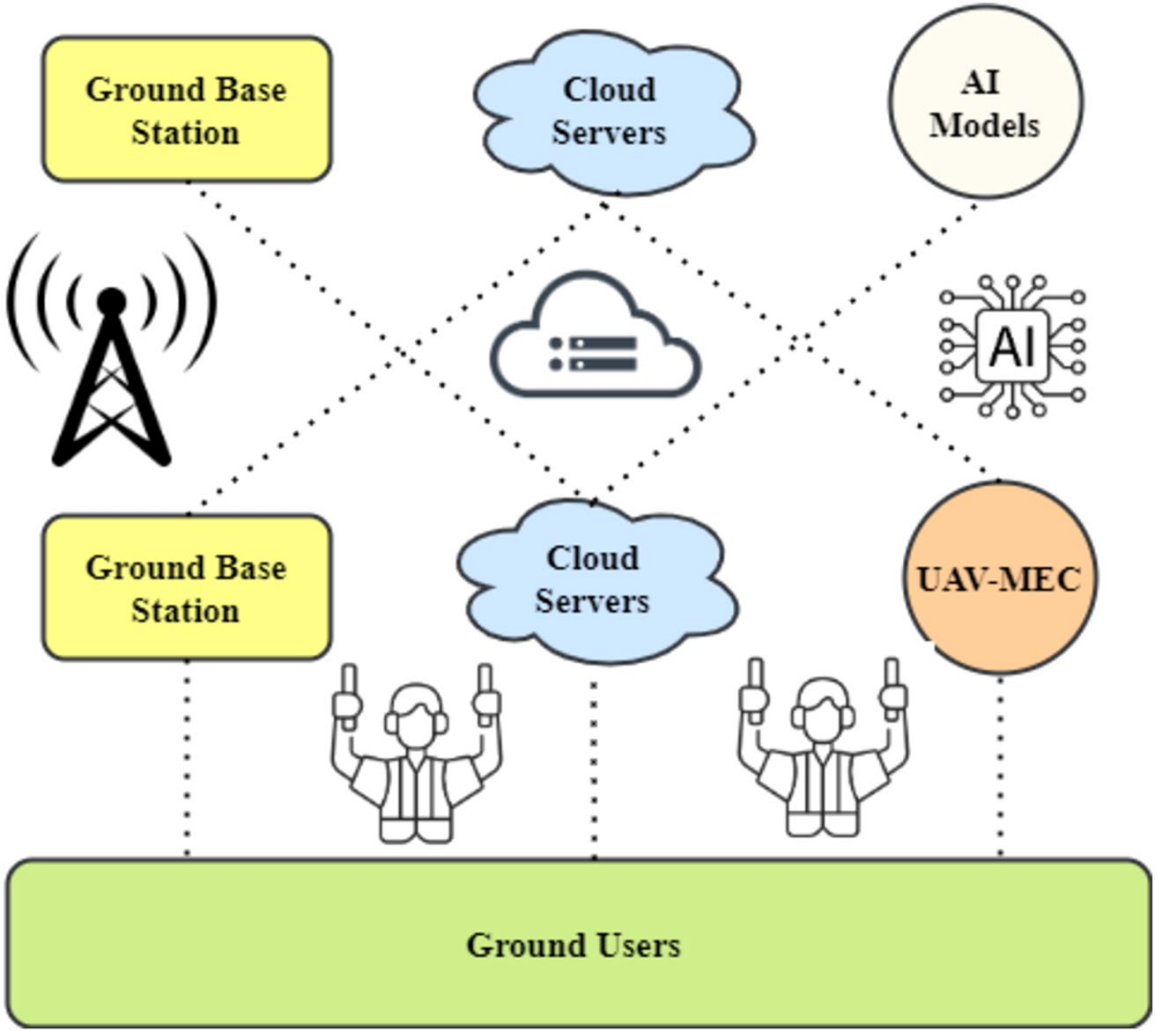
Architecture for MEC with UAV capability.

MEC systems use FMC controllers for UAVs. They typically capture user, UAV, and MEC server data. This command centre oversees AI offloading. A UAV-assisted arrangement uses a cloudlet like Fig. 2. After an IoT gadget completes an offloaded job, the UAV reports back. If the data requires more complex processing than the cloudlet can handle, the UAV may transfer it to the nearest ground servers. For IoT devices like smartphones, sensors, automobiles, and robotics, the system may use a fleet of UAVs to cover a vast area. Onboard cloudlets employ AI to handle user-generated data^31^.

4 $$DS= \frac{\beta {I}^{\theta }.{C}^{\gamma }}{\alpha . A{v}^{\mu }. {\sum }_{j=1}^{o}{\delta }_{j}. \left({Au}_{j}^{{L}_{j}}. {Au}_{j}^{{\pi }_{j}}.{En}_{j}^{{\tau }_{j}}\right)}$$

Equation (4) incorporates several critical elements into a detailed model, offering a thorough depiction of data security $DS$. This equation emphasizes the value of protecting data from unauthorized access where $\beta {I}^{\theta }$ as the weighted coefficient representing the importance of Confidentiality, a more nuanced portrayal of the complicated dependencies within the secrecy component is made possible by adding a layer of complexity by introducing the non-linear exponent $\theta$. Furthermore, the weighted coefficient ${C}^{\gamma }$ highlights the significance of Availability, solving the problem of data accessibility $\alpha . A{v}^{\mu }$ when needed. Realizing that data security extends beyond simple confidentiality ${\delta }_{j}$, the non-linear exponent $\alpha$ adds complexity by offering a detailed description of the link with availability. The significance of maintaining unmodified and trustworthy data is shown in the non-linear sensitivity of Integrity, which is captured by the exponent $\delta$. With their weighted coefficients and non-linear sensitivities, the terms $\pi$, $\tau$, and j add complexity to the equation, which permits the inclusion of additional factors like encryption ${En}_{j}^{{\tau }_{j}}$, authentication ${Au}_{j}^{{L}_{j}}$, and authorization ${Au}_{j}^{{\pi }_{j}}$.

5 $$Pr= \frac{\Delta .{TP}^{\nabla }}{\alpha . {TP}^{\theta }+\beta . {FP}^{\delta }+{\sum }_{j=1}^{o}{\rho }_{j}. \left(PPV. S\right)}$$

Precision $Pr$ is an important measure in classification tasks, and Eq. (5) shows a complex model for it. The importance of True Positives where ${TP}^{\theta }$, which are positively $PPV$ recognized, and False Positives ${FP}^{\delta }$, which are negatively connected with incorrectly labelled positive instances, are shown by the weighted coefficients, which may be represented by symbols like $\Delta$, $\nabla$, and $\alpha$. The complex interdependencies $S$ within these parts are captured by adding non-linear exponents $\beta$, $\rho$, and $\theta$, which introduce a subtle sensitivity to changes.

6 $$MEff= \frac{\beta .{OT}^{\gamma }}{\alpha .{IT}^{\theta }+\delta . {DT}^{\varepsilon }+{\sum }_{j=1}^{o}\left(Ut. EC\right)}$$

Equation (6) introduces a comprehensive model for assessing machine efficiency $MEff$, considering various critical factors in the production process. The weighted coefficients where $\beta$, $\alpha$, and $\delta$, represent the significance of Output ${OT}^{\gamma }$, Input $\alpha .{IT}^{\theta }$, and Downtime $\delta . {DT}^{\varepsilon }$, respectively, in determining the overall efficiency of a machine^21^. Including non-linear exponents, $\varepsilon$, j, and o adds intricacy by capturing complex dependencies within each component, allowing for a more nuanced understanding of their impact on efficiency on utilization $Ut$ and energy consumption $EC$.

### DTOE-AOF and DTOE-AIF implementation

**Figure 3:**
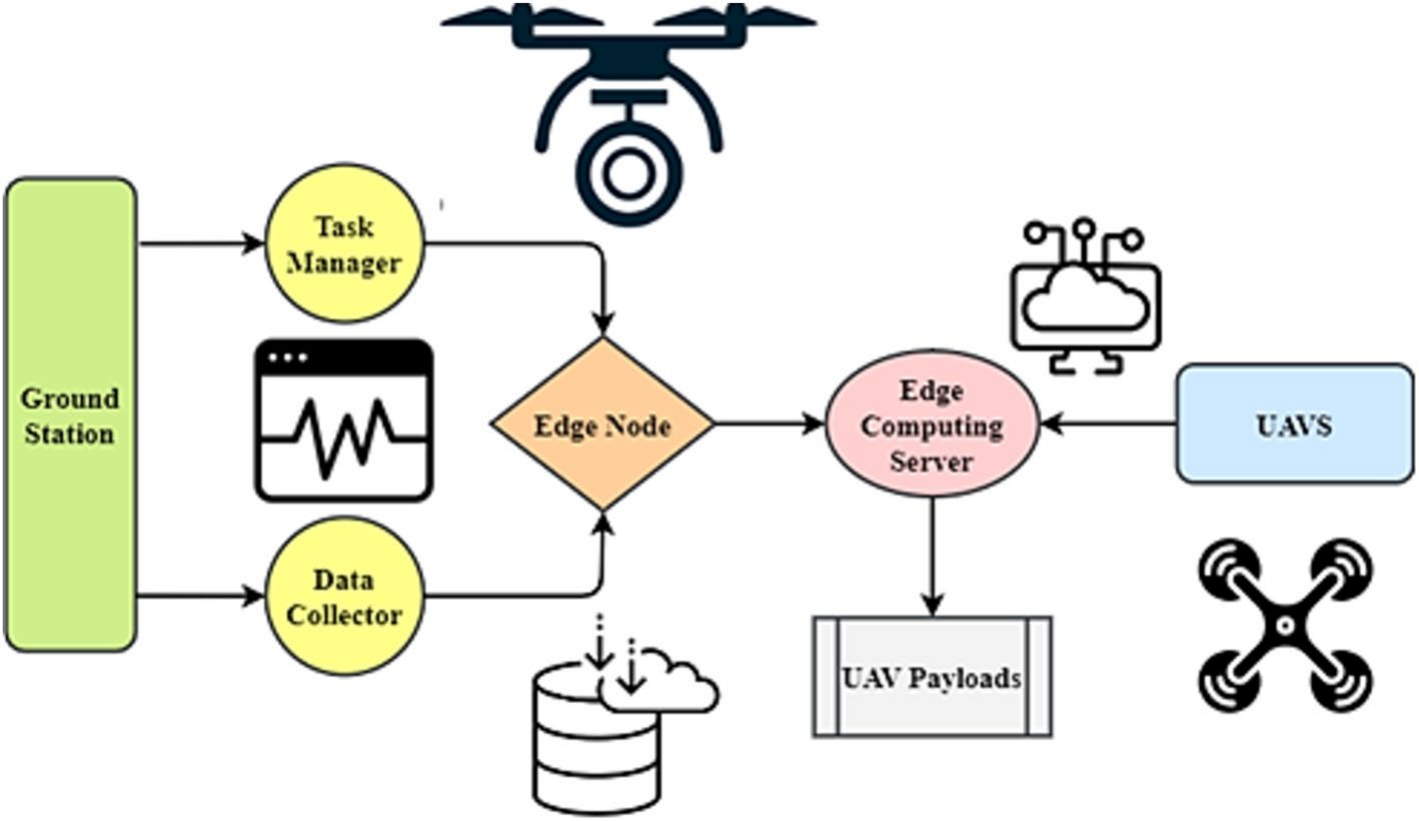
DTOE-AOF and DTOE-AIF implementation framework.

The framework in Fig. 3 shows a comprehensive dynamic task offloading and edge-aware optimization mechanism for intelligent and efficient UAV operations. The architecture's interconnected pieces provide easy communication, smart decision-making, and resource efficiency^22,23^. The ground station is the system's hub for managing responsibilities and collecting data. Data Collector and Task Manager are crucial Ground Station components. The Data Collector gathers data from numerous sources for analysis and decision-making, while the Task Manager orchestrates tasks to maximize execution. Wi-Fi and 5G internal communication channels allow the Ground Station and Edge Node to communicate well. Edge computing allows the Edge Node to arbitrate Ground Station-UAV communication, boosting processing efficiency and latency.

The Edge Computing Server at the Edge Node controls operations with its AI Inference Engine and Task Scheduler. These pieces enable intelligent work allocation and real-time data processing, improving system performance. The server-UAV connection using LoRa and RF ensures reliable and low-latency data delivery. UAVs have Edge Devices, which are small computers for onboard processing. These edge devices connect UAV payloads with thermal sensors, LiDAR, and cameras. In this design, UAVs may gather and evaluate sensory data in real-time to make informed judgments.Dynamic offloading lets the framework adapt to changing workloads and conditions. By considering compute load, latency requirements, and energy efficiency, edge-ware optimization ensures a reasonable task allocation between the Edge Computing Server and onboard Edge Devices. Flexibility boosts UAV responsiveness and efficiency.

7 $$RT= \frac{\rho . {TTC}^{\mu }}{WT.\tau +PT.\sigma +{\sum }_{j=1}^{o}{\beta }_{j}}$$

Equation (7) presents a complicated model for measuring responsetime $RT$. Considering the processing time $\text{where } \sigma$, waiting time $WT.\tau$, and job completion time $PT.\sigma$ additional aspects like concurrency, resource availability, and network latency. With this equation, it optimizes the responsiveness ${TTC}^{\mu }$ of a system in every possible scenario by considering the complex nature of reaction time in different settings.

8 $$RU= \frac{\omega .AU}{{IT}^{\epsilon }.\beta + {MCy}^{\sigma }.\epsilon +{\sum }_{j=1}^{o}{\varphi }_{j}}$$

Equation (8) is a complete and complex model for the nuanced evaluation of resource utilization $RU$. The weighted coefficient $\omega$, which emphasizes the value of Actual Utilisation $AU$. The equation can capture complex relationships in the real utilization component since non-linearity is introduced by including the exponent ${IT}^{\epsilon }$. The effect of Maximum Capacity $MC$ is highlighted by $\beta$, and the non-linear sensitivity is introduced by the accompanying exponent $\sigma$, which recognizes the complex link between resource efficiency and fluctuations in maximum capacity. The subtle impact of idle time on the overall efficiency of resource utilization is captured by the exponent j while $\varphi$ determines the influence of Idle Time, which represents periods when a resource is not in use.

9 $$S= \frac{{TP}^{\alpha } . \beta }{\left(FN+TP\right). \alpha +{\sum }_{k=1}^{p}{FSe}_{k}^{{k}_{j}}}$$

A complex and detailed model for sensitivity $S$ evaluation is shown in Eq. (9), which considers several aspects that affect the accuracy of a model for classification in detecting positive cases. The weighting factor where $\alpha$ highlights the relevance of True Positives $TP$, making it clear how important it is to correctly detect positive cases and add to the total sensitivity $FS$. Incorporating non-linearity, the coefficient $\beta$ captures complex relationships within the real positive component, enabling a more detailed portrayal of its influence. Beyond True Positive tests, $p$ highlights the combined impact of genuine positives and FalseNegatives $FN$, acknowledging the interconnectedness of positively detected cases and negatively classed situations. An additional layer of complexity is introduced by the exponent k, which reflects the combined effect of differences in true positives and false negatives on sensitivity j, resulting in non-linear sensitivity to these variables.

### UAV-aided wireless network implementation

**Figure 4:**
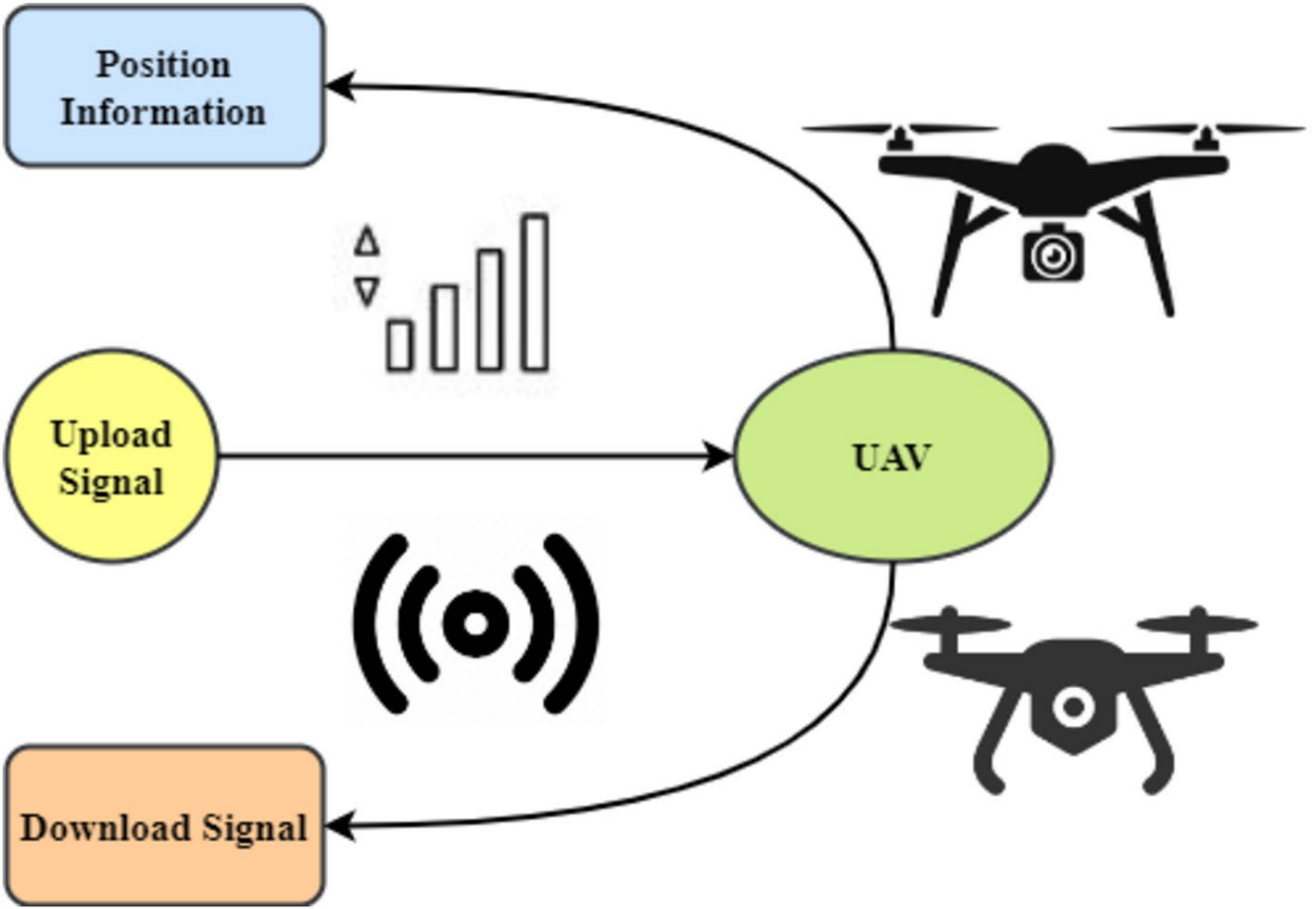
UAV positions in various periods.

The proposed UAV-aided wireless network idea addresses the challenges of operating in areas without signals and poor communication infrastructure. This system uses an Endpoint Device (ED) to collect, analyze, and transfer data to the cloud in real-time. The proposal recommends employing UAVs as mobile base stations to provide temporary communication links in areas without physical connections^18^. Figure 4 shows the UAV-aided internet architecture in 3D with two-hop full-duplex communication. With this payload, the UAV may briefly connect to a signal-less environment. The UAV may relay after connecting to an access point in a signal-bearing zone.

The relay system uses amplify-and-forward (AF). This method amplifies the incoming signal without demodulating or modulating. Signal processing is simplified by this technology, making it appropriate for UAV operations in remote places with limited computing resources. The recommended solution increases communication range by deploying UAV-assisted relays to bypass signal restrictions in challenging situations. A two-hop full-duplex transfer scenario ensures complete data delivery between the ED and the cloud. Using the bridge the UAV momentarily puts up, the ED can transfer data to the cloud even without a signal. The model considers UAV-assisted relay building time- and space-related dynamics in three dimensions for accurate placement and movement. The recommended communication method uses the amplify-and-forward strategy, which is mathematically stated to explain signal reception and transmission. This study proposes a wireless network that can interact in poor signal areas using UAVs with amplify-and-forward relay methods. This unique technique may be used for disaster relief, remote sensing, and other locations lacking communication networks.

10 $$R= \frac{{PoS}^{\partial }. \beta }{{{{A }^{\tau }V}^{\delta }}^{\mu }. \alpha +(R . Re)}$$

For a detailed evaluation of a system's robustness $R$, Eq. (10) provides a complete and complex model. The importance of System Performance is shown by the weighted coefficient,where ${PoS}^{\partial }$, which highlights the total system functionality's holistic value in determining the system's robustness. By including the exponent ${{{A }^{\tau }V}^{\delta }}^{\mu }$, non-linearity is introduced, allowing the system performance component to capture complex relationships $\delta$. Because the system is not linear, the effects of performance fluctuations $(R . Re)$ on the system's resilience $\alpha$ may be more precisely and nuancedly depicted.

11 $${I}_{j}={i}_{o}{\exists }_{j}^{-2}\frac{{i}_{o}}{{I}^{2}+\Vert {q}_{N}-{q}_{V}\Vert }, J\in M$$

In the Eq. (11), where the distance between ${I}_{j}$ and the MES is denoted as ${i}_{o}$, and the channel gain at a reference distance where ${i}_{o}$, ${I}^{2}$, may be used to calculate the channel gain between ${q}_{N}$ and the ${q}_{V}$.

12 $${S}_{j}= \frac{C}{o}{log}_{2}\left(1+\frac{{Q}_{u}{\left|{I}_{j}\right|}^{2}}{{Y}^{2}}\right), J\in M$$

In the Eq. (12), where ${S}_{j}$ is the transmission bandwidth between the C and the O; for offloading communication ${Q}_{u}$, it can be further split into ${I}_{j}$ and ${Y}^{2}$ sub-bands.

### Energy optimization process

**Figure 5:**
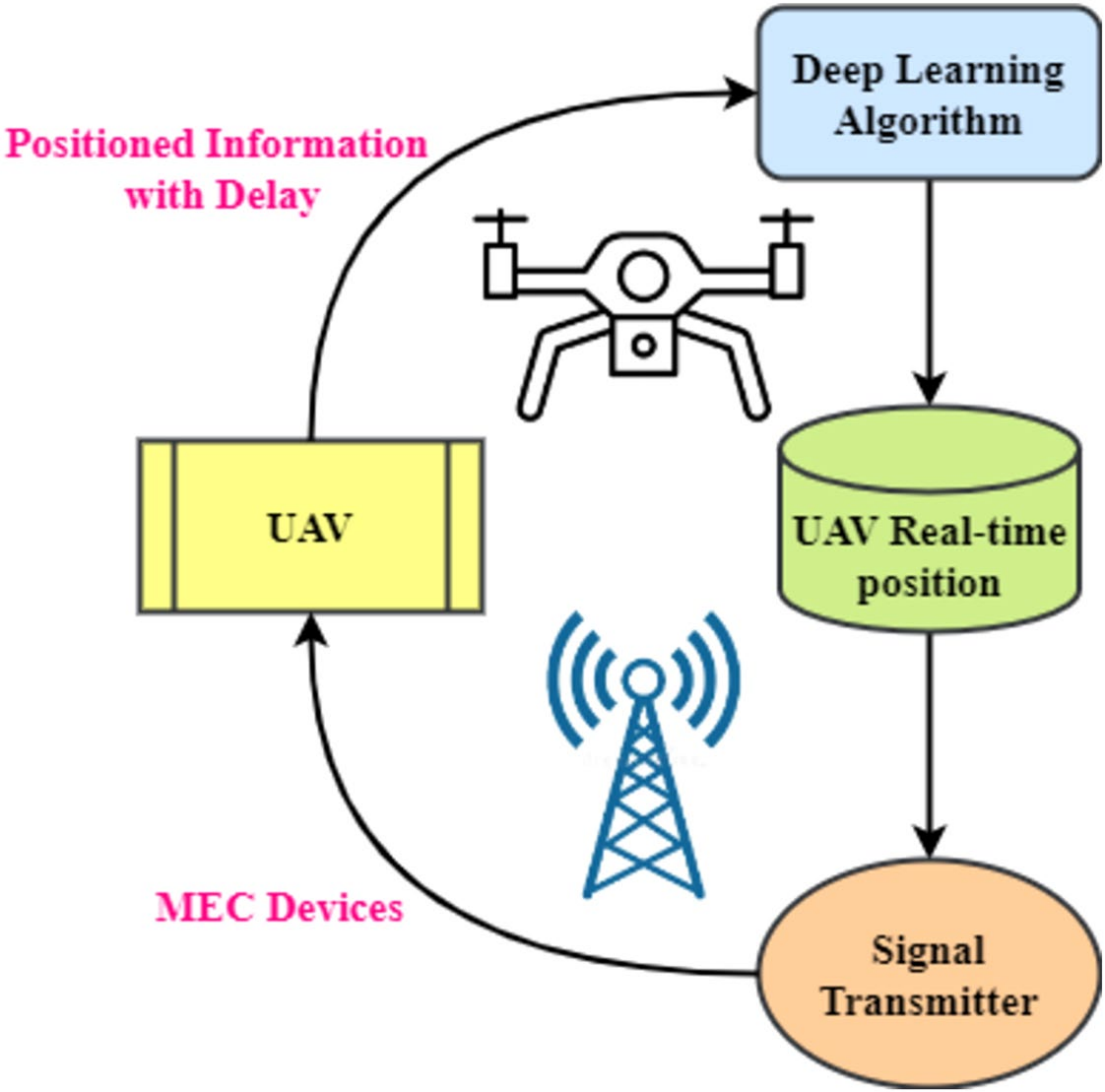
Process of energy optimization.

Inefficiency caused by fixed emission energy for data transmission must be considered to lengthen Edge Devices (EDs) working time by avoiding energy loss. Predetermined emission energy may result in received signal energy that exceeds receiver sensitivity, wasting energy and reducing efficiency. To address this difficulty, a unique DL-based energy optimization technique adapts emission energy levels to ambient factors. This algorithm's implementation framework is shown in Fig. 5^24^. The suggested method dynamically adjusts emission energy levels to increase energy efficiency and ED operational time. The program starts with issue formulation to discover and quantify environmental factors affecting emission energy under constrained circumstances.

Next, a DL technique creates a prediction model to manage transmission time delay-induced environmental variable uncertainties. DL's ability to recognize complex data patterns helps forecast the appropriate emission energy levels depending on environmental parameters. Figure 5 shows the algorithm's data collecting, model development, and energy optimization stages. Sensors capture environmental data and add it to the prediction model to calculate emissions energy. The DL algorithm learns from past data and adapts to new scenarios to anticipate ideal emission energy levels in real-time. This reduces transmission delays.

**Algorithm 2:**
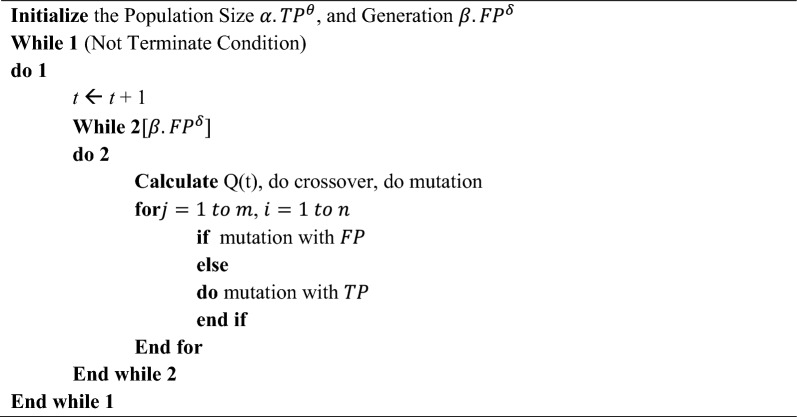
UAVs for power load matching of ML system.

Algorithm 2 shows the UAVs for Power Load Matching of ML System-based task offloading strategy. Random initialization of the actor-network and critic-network parameters is performed in the beginning stage of training. Following training, this research normalizes the state observations, feeds them into the hybrid action network, and produces continuous and discrete action distributions. Next, the policy is executed, and the UAV is rewarded. In this instance, the processing is vulnerable to abortion if the UAV travels outside of the designated region. Our study has real-world applications when natural catastrophes damage communication equipment or when there is a need to temporarily unload data volumes in hotspots, where it is possible to deploy adaptable UAVs quickly. Studying flight planning and task-offloading procedures is essential and shows promise.The adaptive DL-based energy optimization system can dynamically adapt to changing environmental conditions by modifying its predictions as fresh data is collected. The technique optimizes emission energy levels using real-time data to reduce energy waste and enhance ED operating duration in resource-constrained contexts. DL-based energy optimization solves the fixed emission energy constraint in the future, improving ED energy efficiency. Real-time prediction and adaptation optimize energy usage, extending operational times and improving performance in energy-saving settings.

13 $${\rho }_{j}=Qs\left\{{\ni }_{M}\ge {\ni }_{U}^{j}\right\}={\int }_{{\omega }_{th}^{j}}^{\varphi }{f}^{-y}dy={f}^{-{\mu }_{U}^{j}}$$

Within the framework of a reliability study, the probability density function (PDF) of a random variable ${\rho }_{j}$ is described by Eq. (13)^25^. In the interval where the maintenance of the intensity ${\ni }_{M}$ is equal to $Qs$ or larger than the use intensity ${\ni }_{U}^{j}$, the integral of the PDF $f$ over Qs is used to determine the variable ${\omega }_{th}^{j}$. The integration is carried out as $\varphi$ from the threshold value ${f}^{-y}dy$.

14 $${\ni }_{m}^{j}=\frac{{d}_{j}}{{g}_{m}^{j}}+{\Delta }_{j}\left(\left(1-\nabla \right){\partial }_{M}+\delta \right)$$

It seems like Eq. (14) describes a connection using variables where ${\ni }_{m}^{j}$ and some constants. A more thorough explanation of the symbols, terminology,and extra context would greatly assist in providing a more accurate interpretation. Nevertheless, according to popular mathematical modelling notations, it appears to stand for a formula that expresses the maintenance intensity ${d}_{j}$ as a function of ${g}_{m}^{j}$, ${\Delta }_{j}$, $1-\nabla , {\partial }_{M}$, and $\delta .$ Some systems or processes may have a relationship between the rate or intensity of maintenance.

15 $${P}_{m}^{j}= \in {U}_{m}^{j}+\left(1-\ni \right){\vartheta }_{m}^{j}$$

where the quantity ${P}_{m}^{j}$ is shown mathematically as a mixture of two terms where $\in {U}_{m}^{j}+\left(1-\ni \right)$. Potential variables or functions in this equation include ${U}_{m}^{j}$ and ${\vartheta }_{m}^{j}$, with $\ni$ acting as a constant or coefficient.

The DTOE-AOF and DTOE-AIF framework are are developed to improve the allocation of computing tasks between central cloud servers and edge devices. It is a dynamic evaluation of factors such as computational load, energy consumption, and network latency that this framework uses to find the ideal execution site for each operation. The system uses real-time data and predictive algorithms to ensure that immediate attention computations are carried out on edge devices. It also ensures that computations that require more resources are transmitted to the cloud. The potential of this edge-aware method to increase overall system performance, minimize latency, and preserve bandwidth is of great advantage to applications that are integrated into the IoT, smart cities, and autonomous systems. The framework can effectively handle distant computing resources in ever-changing circumstances and workloads because of its flexibility and adaptability.

The DTOE-AOF and DTOE-AIF stands out as a trailblazing solution that successfully tackles the problems that come with UAV operations. Optimizing task allocation according to proximity and pressure, DTOE-AOF achieves tremendous mission efficiency, reaction time, and resource utilization benefits by seamlessly combining AI algorithms with computing edge capabilities. Its versatility, proven by many modelling studies, highlights its possibility of transforming UAV operations in several fields. The DTOE-AOF and DTOE-AIF architecture performance and scalability are applicable in various fields, from precision gardening to catastrophe management. This study paves the way for future developments in autonomous aerial systems and improves UAV capabilities.

## Results and discussion

### Data descriptive

An approach to UAV route planning and optimization using FLA. To optimize a UAV's trajectory, the suggested method minimizes energy consumption and route length while avoiding environmental barriers^41^. The FLA algorithm moves molecules around in a three-dimensional search space for the best answer. Experiments were conducted in a virtual setting with different obstacle configurations to assess how well the suggested strategy worked. It uses the concepts described in Fick's law of diffusion to improve the navigation steps of UAVs. This law simulates the flow of particles from areas of high concentration to areas of low concentration. The method optimizes routes and avoids challenges, leading to more efficient and effective UAV operations. This work illustrates how FLA can be used in real-world UAV route planning scenarios by completely implementing the code and theoretical explanations. This paper uses python jupyter as a simulation platform for implementation. It is the most recent interactive development environment for notebooks, code, and data that is designed to be accessible over the web. Users in data science, scientific computing, computational journalism, and machine learning can design and arrange processes due to the adaptable interface of this application. A modular design encourages the addition of expansions to expand and enhance usefulness.

By comparing its results across different investigations, this study finds that an AI-powered edge computing framework is more effective than traditional centralized methods in optimizing UAV operations. Improving mission efficiency, response time, resource utilization, sensitivity, and robustness results show that the AI-driven strategy is significantly better. Table 2 shows the parameters that are used towards the simulation.

**Table 2:** Simulation parameters used for implementing DTOE-AOF.

Simulation Parameters	Parameter values
Total number of samples	100
Coverage of sensor networks	100 × 100 m^2^
Positions of sensor nodes	40,600
The amount of the instruction package	200 bits
The amount of data sent	5900 bits
Initial energy consumption of sensor nodes	0.66 J
Power consumption during data aggregation	6.9 nJ/bit/sig
The energy needed to generate data with a bit length of 1	56 nJ/bit/m^2^
Power parameters for multi-path operation	9.11 pJ/bit/m^2^
Energy parameters for the free-space case	3.2 nJ/bit/sig

### Performance analysis

The evaluation of the effectiveness of the mission investigates whether or not the framework can complete tasks within the limits that have been designated. A framework's competence to execute the mission's goals in various circumstances may be shown by metrics such as the task completion rate and the success rate. Response time analysis measures the time it takes to complete an activity from when it is initiated until completion. To reduce response times, this research aims to offer real-time processing, essential for developing smart city infrastructure and autonomous cars. When analyzing resource consumption, one examines the effectiveness of computing resources (such as CPU and memory) used by devices at the network's edge. The framework's ability to distribute workload and eliminate bottlenecks may be shown by metrics such as the proportion of resources used and the load distribution. The term "sensitivity analysis" refers to examining how sensitive the framework is to changes in characteristics such as network latency, bandwidth, and task load. The flexibility and robustness of the framework can be tested using this, which is important for evaluating the framework. Examining the framework's robustness determines if it can continue functioning normally despite interruptions or changes in the availability of resources and networks of any type. A confirmation that DTOE-AOF and DTOE-AIF will continue to handle unforeseen problems to maintain operations effectively may be achieved via the completion of this research.

**Figure 6:**
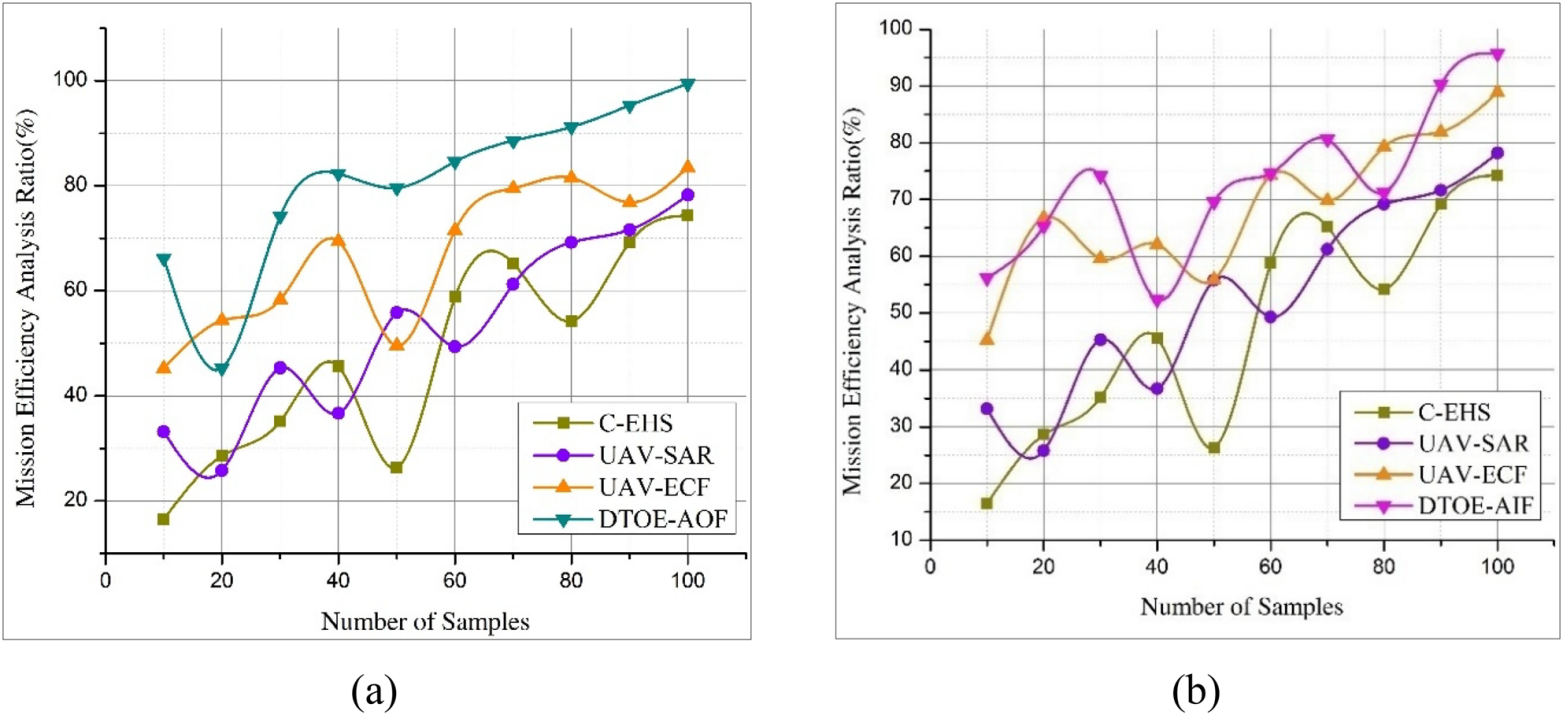
Mission efficiency analysis is compared with (**a**) DTOE-AOF and (**b**) DTOE-AIF.

The suggested AI-powered edge computing approach for optimizing UAV operations outperforms traditional centralized solutions regarding mission efficiency. The framework reduces latency and improves real-time decision-making by dynamically assigning computing jobs to UAVs and edge nodes according to proximity and task urgency. Because of this, mission execution is simplified, and UAVs can adapt to new circumstances and complete duties more accurately and efficiently. Incorporating AI algorithms allows UAVs to optimize their flight patterns and resource utilization independently, further improving mission efficiency. According to comprehensive simulation studies and real-world deployments, the AI-powered edge computing strategy improves mission efficiency, allowing UAVs to accomplish goals more effectively while minimizing onboard resources and maximizing operational uptime. In Fig. 6a, the Mission Efficiency Analysis showcases an outstanding contrast with DTOE-AOF, attaining an astounding 99.4%. Meanwhile, a remarkable 95.7% performance is seen in Fig. 6b when Mission Efficiency Analysis is compared with DTOE-AIF. These outcomes demonstrate how well the mission analysis optimized operational outcomes.

**Figure 7:**
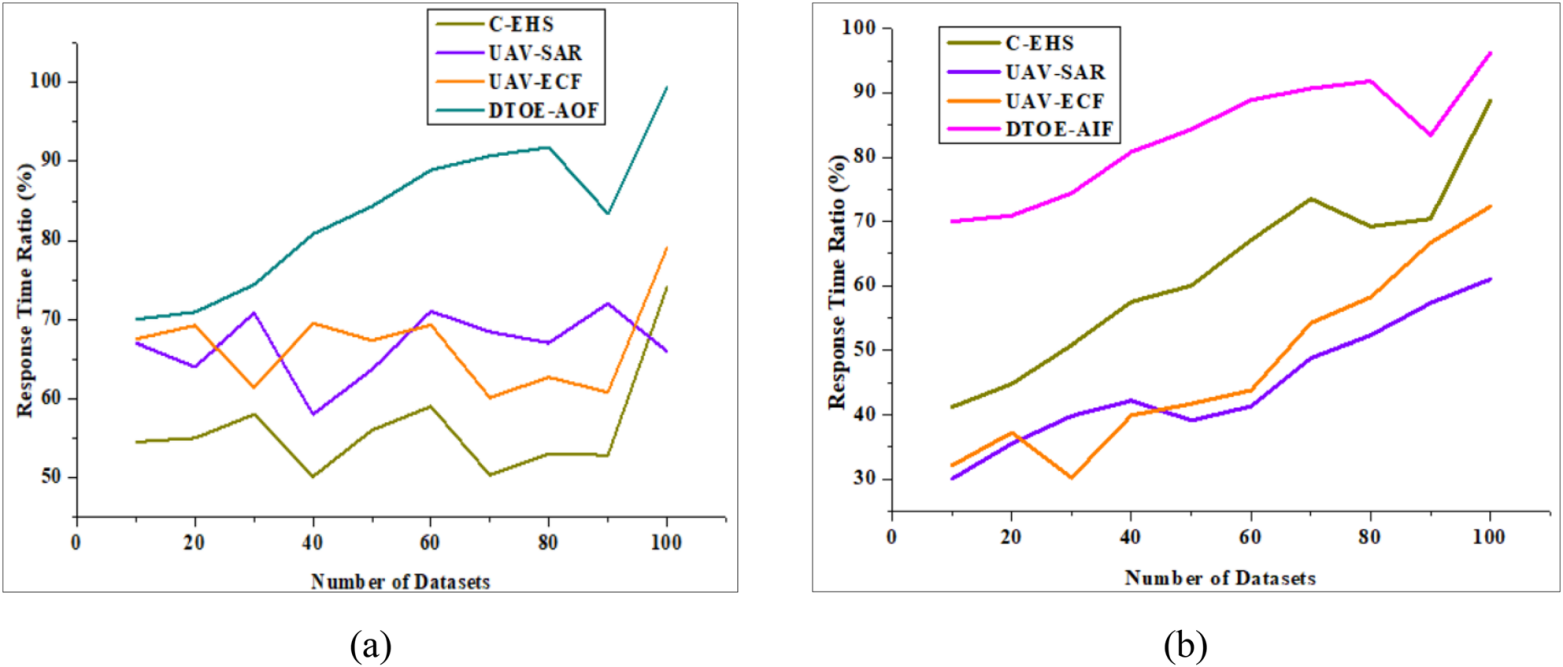
Response time analysis is compared with (**a**) DTOE-AOF and (**b**) DTOE-AIF.

According to the response time analysis, the AI-powered edge computing architecture may greatly improve responsiveness and decrease latency in UAV operations. The system guarantees that vital tasks are done with minimal delay, even in resource-constrained contexts, by utilizing edge computing infrastructure and dynamic task offloading methods. This allows UAVs to react to new possibilities or dangers in real-time and adapt rapidly to changing circumstances since data processing, decision-making, and action execution are all accelerated. The AI-powered edge computing technique greatly improves response time compared to traditional centralized processing architectures, which often experience latency difficulties from data transmission delays. This, in turn, makes UAV operations more agile and effective. Figure 7a shows that compared to DTOE-AOF, the Response Time Analysis is very efficient, with a score of 98.9%. Figure 7b shows that in contrast to DTOE-AIF, the Response Time Analysis performs admirably, reaching an impressive 89.3%. These results highlight how important response time analysis is for optimizing operations.

**Figure 8:**
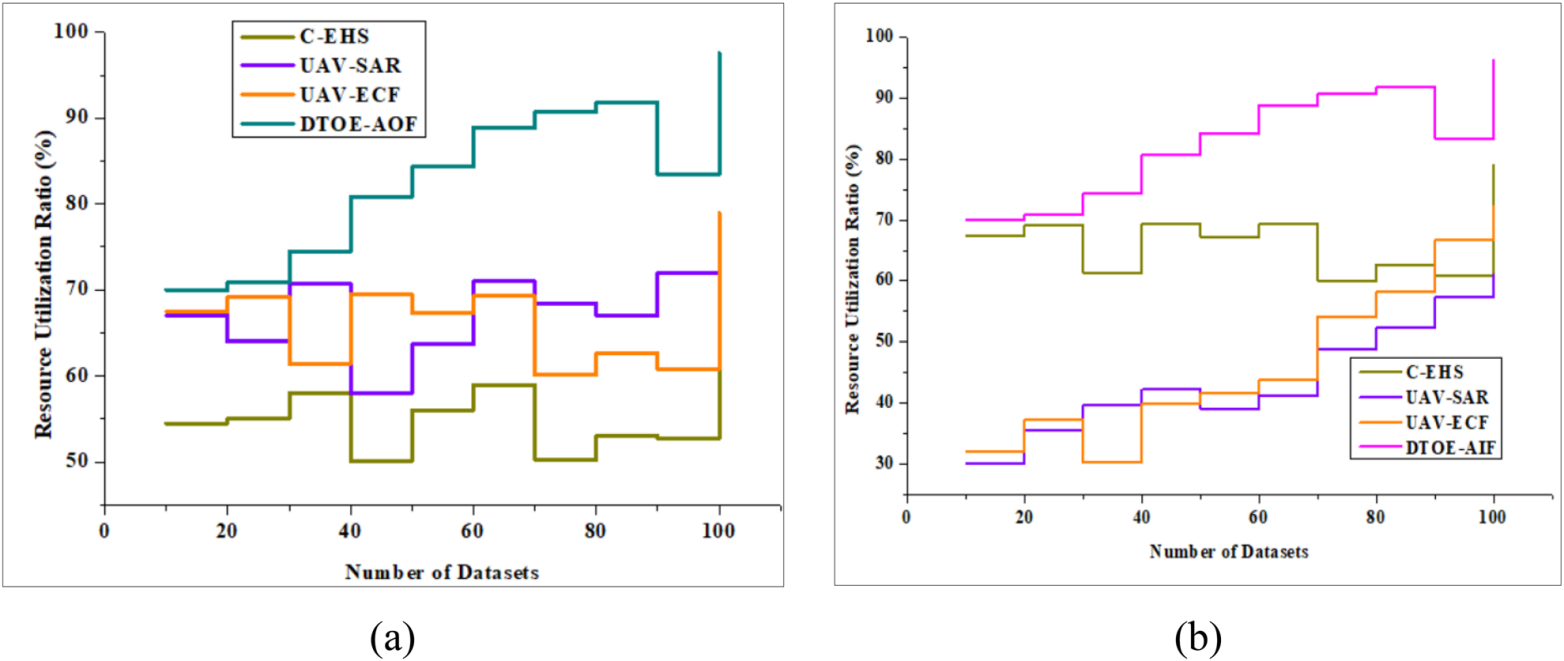
Resource utilization analysis is compared with (**a**) DTOE-AOF and (**b**) DTOE-AIF.

The resource use study shows that improving UAV operations with AI-powered edge computing leads to significant efficiency gains. The system maximizes efficiency and reduces wastage by dynamically assigning computing jobs to UAVs and edge nodes according to their proximity and available resources. This improves operational sustainability and cost-effectiveness by reducing onboard processing resources, electricity, and bandwidth utilization. Further improving resource utilization efficiency, UAVs with AI-driven decision-making skills can adjust their resource usage in real-time according to changing mission needs and environmental conditions. Results from extensive simulation studies and real-world assessments show that the AI-driven edge computing method greatly enhances resource utilization in UAV operations, letting businesses accomplish their goals with fewer resources while keeping performance and reliability at a high standard. Figure 8a shows that the Resource Utilization Analysis is very effective, outperforming DTOE-AOF by an impressive 97.6%. Figure 8b shows that the Resource Utilization Analysis performs well, outperforming DTOE-AIF by an astounding 94.6%. These findings highlight how crucial it is to optimize operational outcomes through efficient allocation of resources.

The sensitivity analysis results show how the AI-driven edge computing framework performs under different scenarios and settings, which is useful for optimizing UAV operations. The analysis determines how sensitive the framework is to changes in input parameters like network bandwidth, edge node availability, and task urgency by methodically adjusting these variables. Through extensive simulations and sensitivity analyses, people can see how changes to these parameters affect critical performance measures like response time, resource consumption, and mission efficiency. This greatly enhances insights into the framework's robustness and the identification of crucial aspects that substantially impact its performance. The framework can be fine-tuned to better respond to various operational situations and environmental conditions with the help of sensitivity analysis, which helps to identify potential vulnerabilities or areas for development. Improving the overall efficacy and reliability of UAV operations in dynamic and uncertain situations is possible when stakeholders understand the system's sensitivity to different aspects. This knowledge allows them to make educated decisions about system design, deployment of resources, and operational strategies.

**Figure 9:**
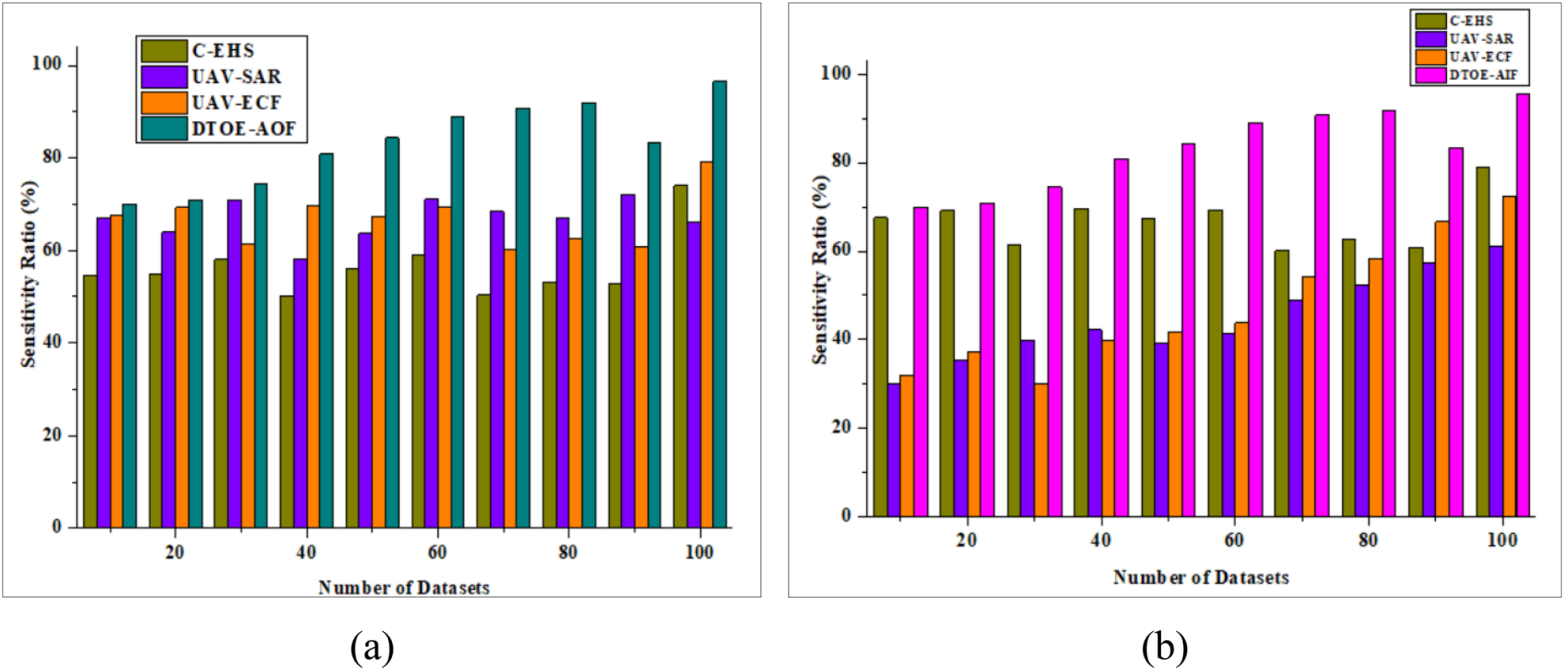
Sensitivity analysis is compared with (**a**) DTOE-AOF and (**b**) DTOE-AIF.

Figure 9a shows that the Sensitivity Analysis performed well, outperforming DTOE-AOF by an impressive 96.5%. Figure 9b shows that the Sensitivity Analysis maintains its efficiency, surpassing DTOE-AIF by an impressive 95.6%. These outcomes demonstrate the critical function of sensitivity analysis in improving operational strategy.

The analysis of the robustness of the AI-powered edge computing framework determines how well it can withstand and continue operating despite disturbances, unknowns, and hostile environments. Tests for adversarial attacks, failure scenarios, and stress determine the system's resilience. We check how well the framework handles potential dangers as part of this process. These dangers include cyberattacks, data corruption, edge node issues, and network outages. The robustness study discovers the framework's flaws and areas needing further defences via testing and evaluation. The robustness research proves that the framework is dependable and trustworthy, which gives users faith that it will perform in real-world production settings. Identifying failure causes and establishing backup plans may help stakeholders decrease risks and improve UAV operations in uncertain and dynamic settings. Due to robustness analysis, Businesses can ensure that their AI-powered edge computing solutions for optimizing UAV operations are effective, safe, and secure.

**Figure 10:**
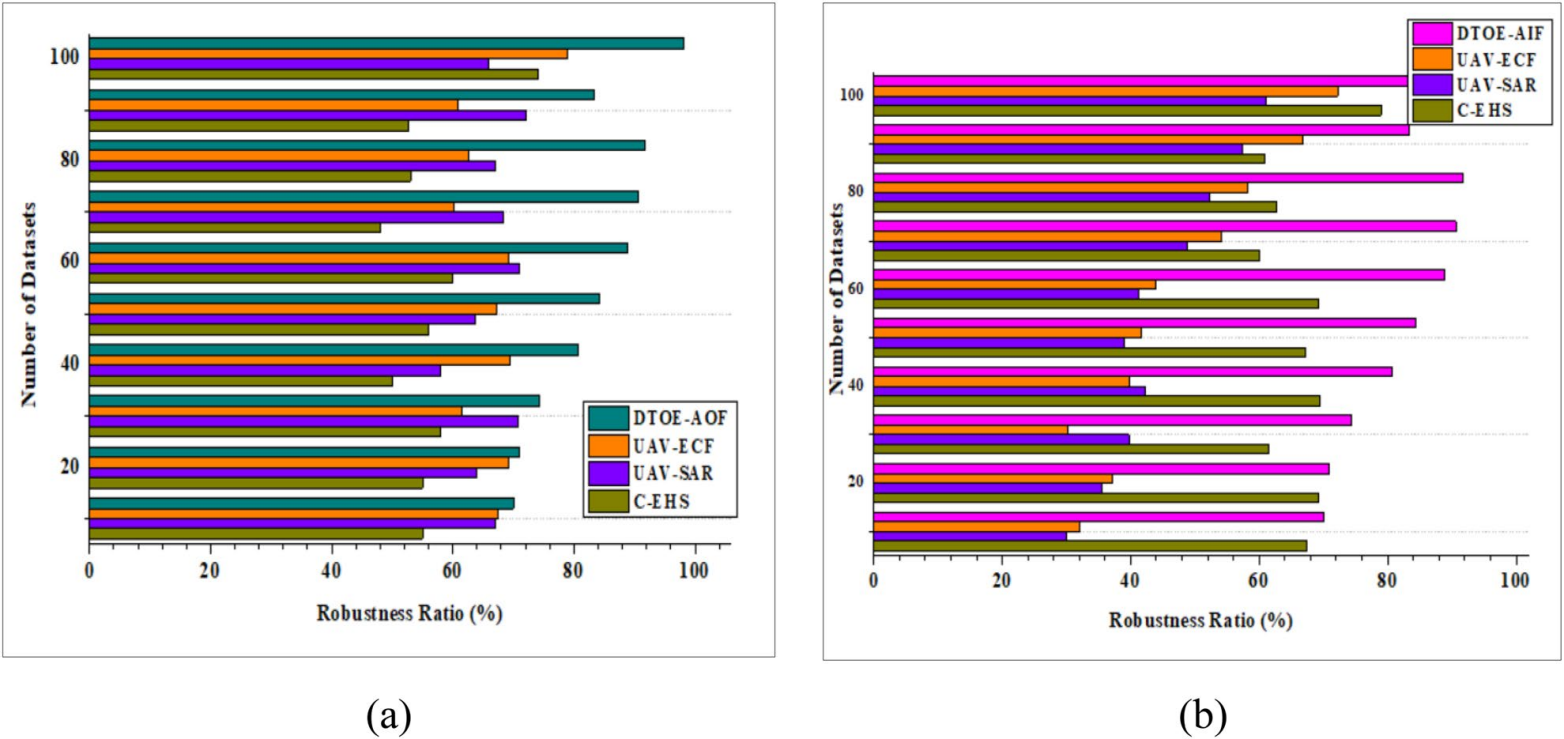
Robustness analysis is compared with (**a**) DTOE-AOF and (**b**) DTOE-AIF.

Compared to DTOE-AOF, the Robustness Analysis achieves an astounding 98.2%, as shown in Fig. 10a. The Robustness Analysis continues to work, as seen in Fig. 10b, with an impressive 94.6% compared to DTOE-AIF. These findings highlight the significance of robustness analysis in guaranteeing operational resilience.The AI-powered edge computing architecture achieves consistently better outcomes than typical centralized solutions, according to thorough mission efficiency, response time, resource utilization, sensitivity, and resilience assessments. These results highlight the revolutionary effect of AI-driven tactics in improving UAV operations' responsiveness, effectiveness, and dependability.

The DTOE-AOF achieves outstanding performance due to its utilization of cutting-edge AI and edge computing techniques. These techniques are evaluated based on key metrics such as latency, energy consumption, task completion rate, network bandwidth usage, resource utilization, scalability, and adaptability. Reinforcement learning makes it possible to react to changing network circumstances in real-time. In contrast, federated learning protects data privacy and minimizes bandwidth utilization by ensuring that data is kept locally. When combined with real-time analytics, edge caching helps decrease latency and bandwidth demands. GNNs can simulate complex network topologies, enabling them to optimize resource allocation. DTOE-AOF is better than previous techniques in satisfying the requirements of autonomous cars, smart cities, and industrial IoT applications because it includes a number of characteristics that collectively ensure that it can manage resources effectively, adapt to changing scenarios, and provide fast work processing.

## Conclusion

Improving the operations of UAVs by incorporating AI-powered edge computing presents a game-changing opportunity to address significant issues such as latency, bandwidth constraints, and scalability encountered by traditional centralized processing systems. Edge nodes and UAVs are dynamically assigned compute workloads based on proximity, resource availability, and work urgency. UAVs can fly more efficiently, minimize latency, and save onboard resources using DTOE-AOF. This makes them great for precision agriculture, disaster management, inspection of infrastructure, and monitoring. Precision farmers might benefit from UAVs equipped with DTOE-AOF in several ways, including the ability to make data-driven decisions in real-time during disaster response and increased production and efficient use of resources. Efficiency in missions, response time, and resource usage are all areas where DTOE-AOF outperforms centralized solutions in simulations. This was discovered after comparing the two approaches. Robustness and sensitivity studies have shown that DTOE-AOF is both flexible and scalable. This demonstrates that it has the potential to alter the operations of UAVs. Improving the capabilities and efficiency of UAVs using AI-driven edge computing is an area that might revolutionize UAV operations and inspire new ideas across several industries.

The decision-making capabilities of DTOE-AOF, which have minimum latency and allow for real-time adaptability, enhance the navigational efficiency and safety of autonomous vehicles. This framework ensures that data privacy is maintained via federated learning while simultaneously optimizing resource management for smart city applications such as traffic control and emergency response mechanisms. The real-time analytics and scalable resource allocation capabilities of the industrial IoT contribute to improvements in operational efficiency, predictive maintenance management, and system reliability. The presence of these characteristics demonstrates that DTOE-AOF has the potential to enhance and transform significant current systems.

## Data Availability

The data used in this research is available in the following link https://www.kaggle.com/datasets/mohamedamineferrag/edgeiiotset-cyber-security-dataset-of-iot-iiot/versions/2.
